# Identification of potential blood biomarkers for Parkinson’s disease by gene expression and DNA methylation data integration analysis

**DOI:** 10.1186/s13148-019-0621-5

**Published:** 2019-02-11

**Authors:** Changliang Wang, Liang Chen, Yang Yang, Menglei Zhang, Garry Wong

**Affiliations:** Cancer Centre, Centre of Reproduction, Development and Aging, Faculty of Health Sciences, University of Macau, Taipa, Macau S.A.R. Macau, China

**Keywords:** Parkinson’s disease, Data integration, DNA methylation, Gene expression

## Abstract

**Background:**

Blood-based gene expression or epigenetic biomarkers of Parkinson’s disease (PD) are highly desirable. However, accuracy and specificity need to be improved, and methods for the integration of gene expression with epigenetic data need to be developed in order to make this feasible.

**Methods:**

Whole blood gene expression data and DNA methylation data were downloaded from Gene Expression Omnibus (GEO) database. A linear model was used to identify significantly differentially expressed genes (DEGs) and differentially methylated genes (DMGs) according to specific gene regions 5′—C—phosphate—G—3′ (CpGs) or all gene regions CpGs in PD. Gene set enrichment analysis was then applied to DEGs and DMGs. Subsequently, data integration analysis was performed to identify robust PD-associated blood biomarkers. Finally, the random forest algorithm and a leave-one-out cross validation method were performed to construct classifiers based on gene expression data integrated with methylation data.

**Results:**

Eighty-five (85) significantly hypo-methylated and upregulated genes in PD patients compared to healthy controls were identified. The dominant hypo-methylated regions of these genes were significantly different. Some genes had a single dominant hypo-methylated region, while others had multiple dominant hypo-methylated regions. One gene expression classifier and two gene methylation classifiers based on all or dominant methylation-altered region CpGs were constructed. All have a good prediction power for PD.

**Conclusions:**

Gene expression and methylation data integration analysis identified a blood-based 53-gene signature, which could be applied as a biomarker for PD.

**Electronic supplementary material:**

The online version of this article (10.1186/s13148-019-0621-5) contains supplementary material, which is available to authorized users.

## Background

Parkinson’s disease (PD) is the second most common neurodegenerative disease, following Alzheimer’s disease. PD mainly affects the patient’s motor system, the symptoms of which include tremor, rigidity, and slowness of movement [1]. PD was first mentioned in 1817 by Doctor James Parkinson; however, there still remains no cure for PD today [2]. A wide body of literature currently suggests that genetic or environmental factors can lead to PD [3]. But knowledge concerning the detailed processes governing the initiation and progression of PD is still unknown and remains a key obstacle on the road of PD treatment. Development of robust and accurate biomarkers would greatly facilitate the early detection and identification of biological features of PD. Therefore, it is urgent to identify potential biomarkers for PD.

Currently, brain imaging of the nigrostriatal dopamine system has been used as a biomarker for early disease along with cerebrospinal fluid analysis of α-synuclein. However, these methods remain costly or are invasive [4]. Blood biomarkers are easier to obtain, much cheaper, and less invasive [5], and some researchers have demonstrated that some biomarkers for PD exist in blood [6–8].

In past years, the abnormal expression or altered epigenetic modification of PD-associated genes including PARK1-15, LRRK2, SNCA, MAPT, and GBA were suggested to be associated with PD pathology [9, 10]. For example, α-synuclein, encoded by the SNCA gene, is a major component of Lewy bodies, which are an already known neuropathological feature of PD [11]. Overexpressed α-synuclein was verified in association with pathogenesis of PD [12]. Abnormal accumulation of α-synuclein and the formation of Lewy bodies could trigger the body’s inflammatory response, which was previously thought to be the result of PD but recently has been verified as one of the causes [13]. In addition, hypo-methylated α-synuclein DNA was observed in PD patients [14]. Methylation of cytosines, the key epigenetic modification of DNA in eukaryotes, is associated with inhibition of gene expression [15]. Therefore, hypo-methylation might be one of the causes of over-expression of PD-associated genes. Previous studies mainly focused on studying methylation of the promoter region [16–18]. However, some PD-associated 5′—C—phosphate—G—3′ (CpGs) are located at other gene regions [19, 20].

In our study, we measured the gene methylation level according to CpGs of different regions. In addition, we integrated DNA methylation data and gene expression data to identify molecules and their epigenetic modifications underlying PD. We found that there are some genes that are both abnormally expressed and have altered methylation in PD patients compared to healthy controls. Notably, over 90% of the genes with these overlaps are both hypo-methylated and upregulated genes. Then, we used the hypo-methylated and upregulated genes to construct classifiers based on gene expression data and methylation data, which can distinguish PD patients from healthy controls.

## Methods

**Fig. 1:**
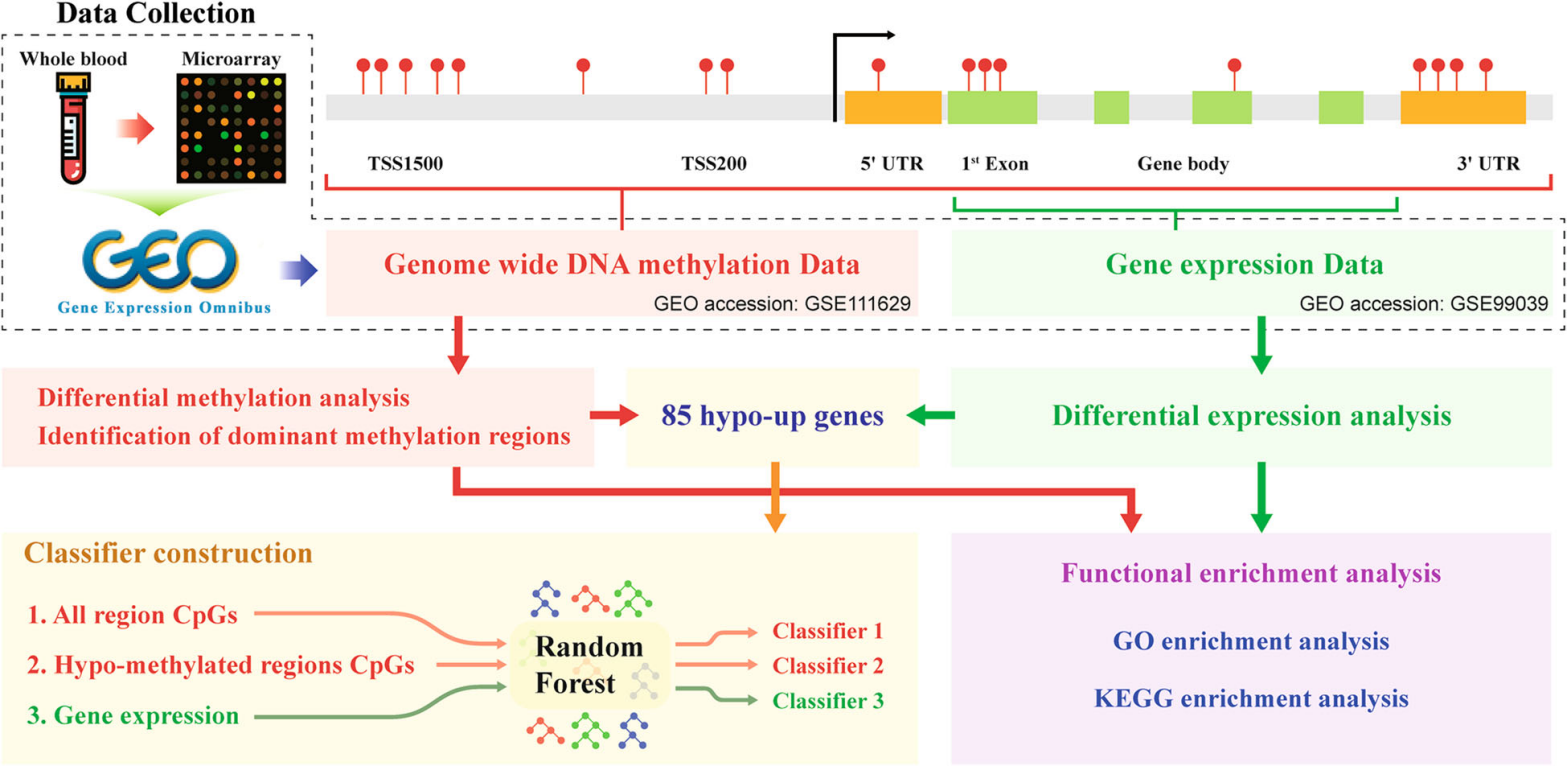
Flowchart of the analysis process

Our methods include the following steps: data collection, differential expression analysis, differential methylation analysis, dominant hypo-methylated region identification , enrichment analysis, and classifier construction. The workflow is shown in Fig. 1.

### Data collection

Gene expression data (GSE99039) [21] from 233 healthy controls and 205 PD patients were downloaded from Gene Expression Omnibus (GEO) database [22]. The data were measured by the Affymetrix Human Genome U133 Plus 2.0 Array, were preprocessed using RMA, and the data were log2 transformed and quantile normalized. In addition, one Alzheimer’s disease (AD) associated blood gene expression dataset (GSE85426) including 90 AD samples and 90 healthy controls, and one Huntington’s disease (HD) associated blood gene expression dataset (GSE51799) [23] including 91 HD patients and 33 healthy controls were used to validate the PD specificity of our classifier. We downloaded the expression matrix and platform information using R package “GEOquery” [24].

Genome wide DNA methylation data (GSE111629) [19], containing data from 335 PD patients and 237 healthy controls in whole blood samples, were downloaded from GEO database [22]. The data were measured by the Illumina Infinium 450 k Human DNA methylation Beadchip, and the raw methylation data (beta values) were preprocessed using the background normalization method from the Genome Studio software. We downloaded the normalized methylation data from GEO database (https://www.ncbi.nlm.nih.gov/geo/download/?acc=GSE111629&format=file&file=GSE111629%5FPEGblood%5F450kMethylationDataBackgroundNormalized%2Etxt%2Egz) and downloaded the platform information using R package “GEOquery” [24].

### Differential expression analysis

A linear model was used to assess differential expression between PD patients and healthy controls using R package named “limma” [25]. Benjamini and Hochberg’s method (BH) was used to control the false discovery rate across all the genes. We identified the significantly differentially expressed gene using threshold BH adjusted *p* value < 0.05 and absolute log2FC > 0.1.

### Differential methylation analysis

One of the most widely used techniques to measure DNA methylation is the Illumina Infinium HumanMethylation450 BeadChip array, which covers approximately 450,000 CpG sites at different gene regions including TSS1500, TSS200, 5′UTR, 1stExon, body, and 3′UTR. TSS1500 refers to 200–1500 bases upstream of the transcriptional start site (TSS). TSS200 means 0–200 bases upstream of TSS. 5′UTR stands for the 5′ untranslated region, defined as the region between the TSS and the ATG start site. 1stExon is short for the first exon of the gene. Body is the region between ATG start site and stop codon. 3′UTR is short for 3′untranslated region that is between the stop codon and poly-A tail. At each CpG site, methylation is quantified by the beta value *b* = *M*/(*M* + *U* + *a*), (where *M* > 0, *U* > 0, *a* ≥ 0), where *M* and *U* represent the methylated and unmethylated signal intensities, respectively. With attention that both M and U are small, *a* is usually set as 100 to stabilize beta values [26]. In our study, we measured region-specific gene methylation level using the average beta value of the CpGs in the region. Meanwhile, we also measured the methylation level of a specific gene using the average of beta value of the CpGs in all gene regions. *M* value is another value to measure gene methylation level, which is a logit transformation of the beta value. *M* value provides much better performance in terms of detection rate and true positive rate for both highly methylated and unmethylated CpG sites [27]. We converted beta value to *M* value. Then, we used linear model to measure the methylation difference between PD patients and healthy controls. In addition, as beta value has a more intuitive biological interpretation than *M* value [27], we also calculated the delta of beta value between PD patients and healthy controls for each gene. In our study, we used both *M* value and beta value to determine the differentially methylated genes or intergenic CpG sites. We calculated the 10 quantile of delta beta value of all genes and all intergenic CpG sites, respectively, then we used the genes and intergenic CpG sites with delta beta value < 1/10 quantile or > 8/10 quantile and BH adjusted *p* value < 0.05 as the significantly differentially methylated genes or intergenic CpG sites between PD patients and healthy controls. The conversion between beta value and *M* value was fulfilled by R package named “lumi” [28]. Differential analysis was implemented by R package “limma”. The Circos plot was implemented by R package “RCircos” [29]. The chromosome distribution plot was implemented by R package “chromoMap” [30].

### Identification of dominant hypo-methylated regions

Firstly, we found the gene region with the smallest delta of beta value (PD compared to control). Then, if the difference between the delta of beta value of another region and the smallest delta of beta value is smaller than 0.005, we regarded the region as one of the dominant hypo-methylated regions.

### Enrichment analysis

Gene ontology (GO) [31, 32], Kyoto Encyclopedia of Genes and Genomes (KEGG) [33] pathway enrichment analysis, and the illustration of enrichment results were implemented using R package “clusterProfiler” [34]. We performed GO term enrichment analysis under the following three sub-ontologies: biological process (BP), molecular function (MF), and cellular component (CC).

### Classifier construction

The random forest algorithm was used to construct classifiers to distinguish PD patients from healthy controls based on gene expression data and gene methylation data, separately. Leave-one-out cross validation method was used to assess the performance of the classifier. For gene expression classifier, we used log2 transformed expression data to train the classifier. For gene methylation classifier, we firstly used average beta value of all region CpGs to measure gene methylation level and train the classifier. Then, we used the average beta value of the dominant methylation-altered region CpGs to measure gene methylation level and train another methylation classifier. The random forest algorithm is implemented by R package “randomForest” [35] and “party” [36, 37]. We used the area under the curve (AUC) of receiver operating characteristic curve (ROC) to measure the quality of the classifier, which is implemented by R package “verification” (https://cran.r-project.org/web/packages/verification/index.html).

## Results

### Identification of blood-based DEGs in PD

To identify the differentially expressed genes (DEGs) in the blood of PD patients compared to healthy controls, one blood microarray dataset (GSE99039) with the biggest sample number (203 PD patients and 233 healthy controls) has been analyzed using the linear modeling approach. We identified 1045 significantly DEGs (adjusted *p* value < 0.05 and log2FC > 0.1) in blood of PD compared to healthy controls, in which 108 genes are downregulated and 937 genes are upregulated (Additional file 1: Table S1). In order to compare our differential results with the results from the original paper, we have made a table including the 100 most differential probes that are mapped to 87 genes from the original paper with logFC, *p* value, and BH adjusted *p* value from our analysis (Additional file 1: Table S2). As the table shows, there are 38 probes that are mapped to 31 genes that satisfy our threshold. Then, we listed the top enriched GO terms and KEGG pathways in Table 1 and listed all enriched terms in (Additional file 1: Table S3). As Table 1 shows, downregulated genes are significantly associated with a molecular function (MF)-named structural constituent of cytoskeleton, which plays important roles in dopaminergic neurotransmission [38]. Upregulated genes are associated with Phagosome and Lysosome pathways, which play important roles in mis-folded protein degradation [39], which is a process associated with PD pathogenesis [40]. In addition, these dysfunctional genes are also associated with neutrophil mediated immunity, granulocyte activation, and neutrophil activation, which belong to the innate immune system, and the activation of innate immune system has been verified in association with or in response to Lewy body formation [41, 42]. These results reveal that circulating DNA methylation may reflect the status of PD.

**Table 1:** List of top enriched GO terms and KEGG pathways of DEGs

Direction	Terms	ID	Description	*p* value	*p* adjust
Upregulated	BP	GO:0036230	Granulocyte activation	2.18E−46	7.79E−43
Upregulated	BP	GO:0002446	Neutrophil mediated immunity	4.19E−46	7.79E−43
Upregulated	BP	GO:0042119	Neutrophil activation	4.90E−46	7.79E−43
Upregulated	BP	GO:0002283	Neutrophil activation involved in immune response	1.10E−45	1.05E−42
Upregulated	CC	GO:0030667	Secretory granule membrane	2.91E−23	1.57E−20
Upregulated	CC	GO:0042581	Specific granule	2.18E−21	5.87E−19
Upregulated	CC	GO:0070820	Tertiary granule	3.46E−19	6.21E−17
Upregulated	CC	GO:0034774	Secretory granule lumen	2.28E−15	3.06E−13
Upregulated	CC	GO:0060205	Cytoplasmic vesicle lumen	5.00E−15	5.09E−13
Upregulated	MF	GO:0003779	Actin binding	1.70E−08	1.37E−05
Upregulated	MF	GO:0031267	Small GTPase binding	1.58E−06	3.59E−04
Upregulated	MF	GO:0019902	Phosphatase binding	1.59E−06	3.59E−04
Upregulated	MF	GO:0051020	GTPase binding	1.78E−06	3.59E−04
Upregulated	KEGG	hsa04380	Osteoclast differentiation	4.91E−12	1.32E−09
Upregulated	KEGG	hsa04650	Natural killer cell mediated cytotoxicity	1.44E−11	1.94E−09
Upregulated	KEGG	hsa04142	Lysosome	6.26E−09	5.61E−07
Upregulated	KEGG	hsa04612	Antigen processing and presentation	8.95E−09	6.02E−07
Upregulated	KEGG	hsa04145	Phagosome	3.97E−08	2.14E−06
Downregulated	MF	GO:0005200	Structural constituent of cytoskeleton	1.42E−04	3.5E−02

*BP* biological process, *MF* molecular function, *CC* cell component, *KEGG* Kyoto Encyclopedia of Genes and Genomes

### Identification of blood-based differentially methylated intergenic CpGs in PD

**Fig. 2:**
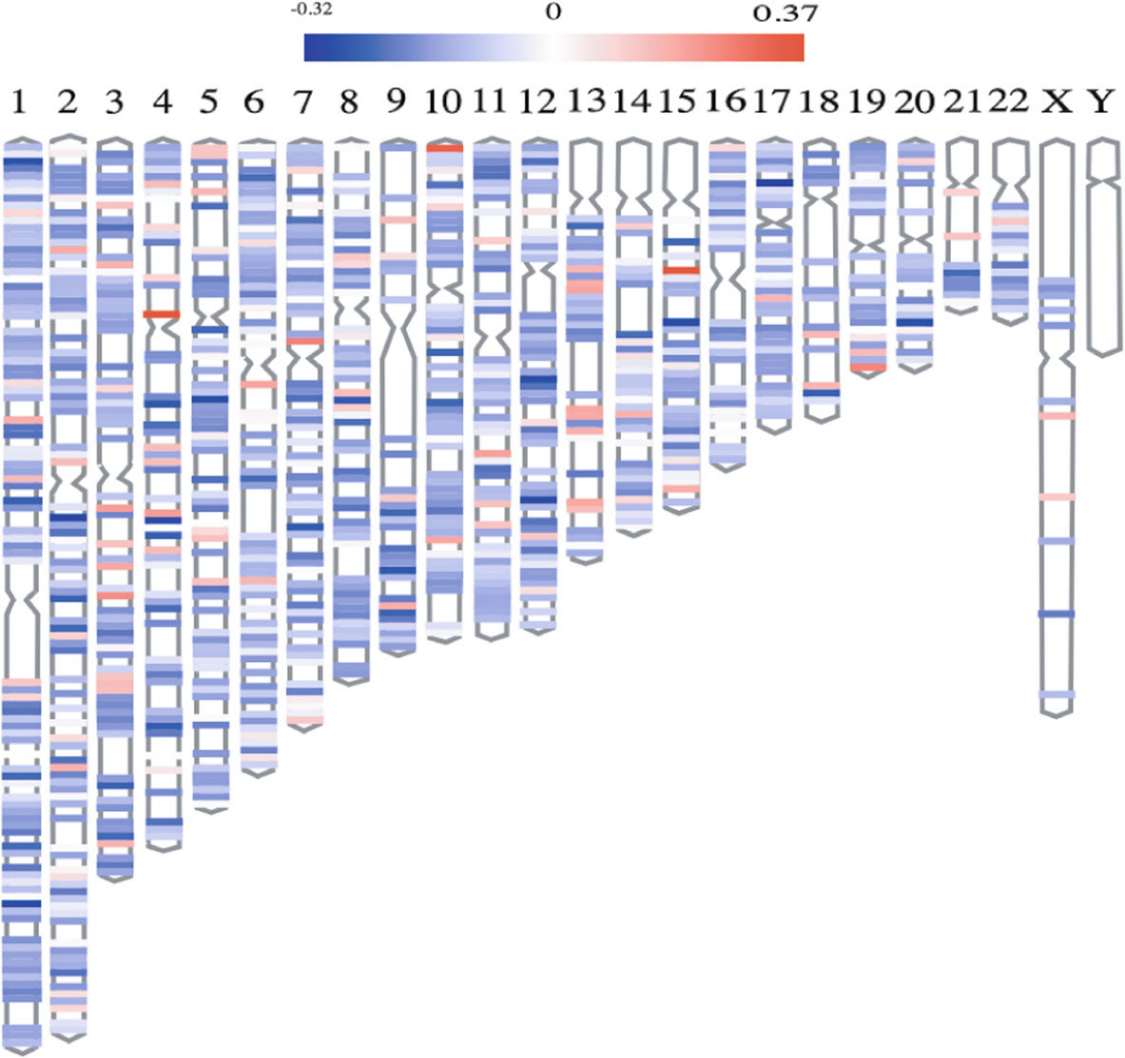
Chromosome distribution of differentially methylated intergenic CpGs. The plot displays the distribution of differential intergenic CpG sites at 22 autosomes, the X chromosome, and the Y chromosome. The region in red is a hyper-methylated region, and the region in blue is a hypo-methylated region. The value is the logFC of the *M* value between PD patients and healthy controls

Intergenic CpG sites account for about 25% of CpGs of Illumina Infinium HumanMethylation450 BeadChip array. The ENCODE Consortium has already identified some enhancer-associated intergenic CpGs and distal promotor-associated CpGs using informatics [43]. In addition, some intergenic CpGs were experimentally determined DMRs, which include some cancer-specific and reprogramming-specific differentially methylated genes (DMGs) [44]. These findings revealed that there might be some differentially methylated intergenic CpGs in blood for PD. Then, we identified 4162 differentially methylated intergenic CpGs based on linear modeling approach and the delta of beta value (Additional file 1: Table S4). Notably, approximately 80% of these differential intergenic CpGs are hypomethylated in PD patients compared to healthy controls. Figure 2 shows the chromosome distribution of these differential intergenic CpGs. Table 2 shows the number of enhancer-associated and non-enhancer-associated intergenic CpGs in differential intergenic CpGs and non-differential intergenic CpGs. The chi-square test was used to test whether there was a significant difference of enhancer-associated CpG percentage between differential group and non-differential group. Interestingly, we found that the percentage of enhancer-associated CpGs in these differential intergenic CpGs is significantly higher than that in non-differential intergenic CpGs (*p* value < 2.2e–16), which implies that there might be some differentially methylated intergenic CpGs in distal enhancers that could regulate gene expression and play some roles in PD initiation or progression.

**Table 2:** Enhancer-associated CpG number table

Group	Number of enhancer-associated intergenic CpGs	Number of non-enhancer-associated intergenic CpGs
Differential intergenic CpG	2100	2061
Non-differential intergenic CpG	36,832	79,575

### Identification of blood-based DMGs in PD based on different region CpGs

**Fig. 3:**
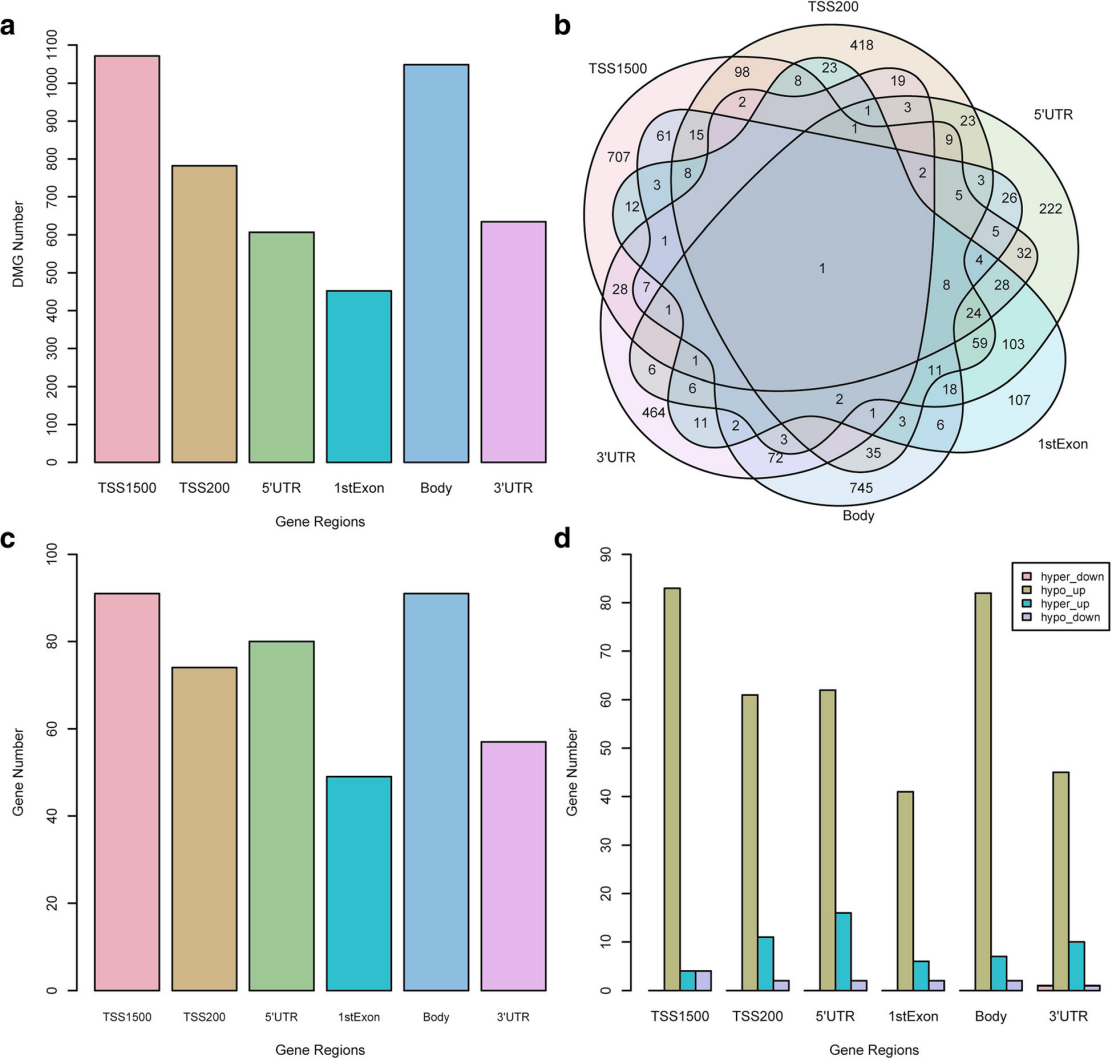
Integration analysis results of DMGs based on different region CpGs and DEGs. **a** Barplot for different region DMGs. The *y*-axis is the number of DMGs. The *x*-axis labels different gene regions: TSS1500, TSS200, 5′UTR, 1stExon, body, and 3′UTR. TSS1500 refers to 200–1500 bases upstream of the transcriptional start site (TSS). TSS200 means 0–200 bases upstream of TSS. 5′UTR stands for the 5′ untranslated region located between the TSS and the ATG start site. 1stExon is short for the first exon of the gene. Body is the region between ATG start site and stop codon. 3′UTR is short for 3′ untranslated region that is between stop codon and poly-A tail. **b** Venn plot for different region DMGs. The numbers on the diagram represent the DMG numbers in a specific region or multiple regions. Each region name is labeled beside the region circle. **c** Barplot for the overlap genes between DEG and different region DMGs. The *y*-axis is the number of overlap genes. The *x*-axis labels different gene regions: TSS1500, TSS200, 5′UTR, 1stExon, body and 3′UTR. **d** Barplot of four groups that overlap in each region. Hyper-down represents hyper-methylated and downregulated genes. Hypo-up represents hypo-methylated and upregulated genes. Hyper-up represents hyper-methylated and upregulated genes. Hypo-down represents hypo-methylated and downregulated genes. The *y*-axis is the number of genes

CpG sites are distributed at different regions of a specific gene including TSS1500, TSS200, 5′UTR, 1stExon, body, and 3′UTR. However, the functions of the methylation of different gene regions remain unclear, so we measured the gene methylation level based on different gene region CpGs, respectively. Then, we identified significantly differentially methylated genes (DMGs) based on the linear modeling approach and the delta of beta value (Additional file 1: Table S5). As Fig. 3a shows, there are more than 1000 DMGs at TSS1500 or body region. However, there are only ~ 400 DMGs at 1stExon region. As shown in the Venn diagram Fig. 3b, over 50% of DMGs at TSS1500, TSS200, body, and 3′UTR are region-specific DMGs. While, DMGs at 5′UTR and 1stExon are mostly shared with other regions. Notably, there are over 250 overlap DMGs between the two regions and 103 are unique overlap DMGs, which is almost equal to the number (107) of 1stExon-specific DMGs. The results reveal that 5′UTR and 1stExon might have a relatively similar methylation alteration level in PD. In addition, PRTN3 is a hypo-methylated gene shared by all gene regions, which is associated with cytokine signaling in the immune system and response to elevated platelet cytosolic Ca^2+^ pathway.

The top enriched BP terms for each region DMGs are listed in Table 3, and all enriched BP terms are listed in supplementary material (Additional file 1: Table S6). As Table 3 shows, BP terms enriched by DMGs based on different gene region CpGs are hypo-methylated genes in TSS1500, TSS200, 5′UTR, 1stExon, and body and are associated with neutrophil mediated immunity, granulocyte activation, neutrophil activation, etc., which play important roles in the formation of Lewy body [41, 42]. Notably, hyper-methylated genes in TSS1500, 5′UTR, 1stExon, and body are all associated with T cell activation. Sulzer and coworkers have established that T cells from patients with PD can recognize α-synuclein peptides [45].

**Table 3:** List of top enriched GO-BP terms for each region DMGs

Direction	Region	ID	Description	*p* value	*p* adjust
Hyper-methylated	TSS1500	GO:0031295	T cell costimulation	1.03E−09	1.40E−06
Hyper-methylated	TSS1500	GO:0031294	Lymphocyte costimulation	1.44E−09	1.40E−06
Hyper-methylated	TSS1500	GO:1903037	Regulation of leukocyte cell-cell adhesion	6.11E−08	3.95E−05
Hyper-methylated	5′UTR	GO:0050851	Antigen receptor-mediated signaling pathway	3.83E−08	9.36E−05
Hyper-methylated	5′UTR	GO:0050852	T cell receptor signaling pathway	3.88E−07	4.74E−04
Hyper-methylated	5′UTR	GO:0002768	Immune response-regulating cell surface receptor signaling pathway	9.75E−06	7.16E−03
Hyper-methylated	5′UTR	GO:0042110	T cell activation	1.17E−05	7.16E−03
Hyper-methylated	1stExon	GO:0050851	Antigen receptor-mediated signaling pathway	2.06E−07	3.81E−04
Hyper-methylated	1stExon	GO:0050852	T cell receptor signaling pathway	4.35E−06	2.86E−03
Hyper-methylated	1stExon	GO:0042110	T cell activation	4.64E−06	2.86E−03
Hyper-methylated	Body	GO:0042110	T cell activation	6.82E−08	1.66E−04
Hyper-methylated	Body	GO:0030217	T cell differentiation	3.62E−07	4.41E−04
Hyper-methylated	Body	GO:0030098	Lymphocyte differentiation	5.90E−07	4.79E−04
Hyper-methylated	3′UTR	GO:0002791	Regulation of peptide secretion	5.12E−05	4.85E−02
Hyper-methylated	3′UTR	GO:0002700	Regulation of production of molecular mediator of immune response	6.12E−05	4.85E−02
Hyper-methylated	3′UTR	GO:0045429	Positive regulation of nitric oxide biosynthetic process	1.02E−04	4.85E−02
Hypo-methylated	TSS1500	GO:0002283	Neutrophil activation involved in immune response	2.28E−21	3.68E−18
Hypo-methylated	TSS1500	GO:0043312	Neutrophil degranulation	2.28E−21	3.68E−18
Hypo-methylated	TSS1500	GO:0002446	Neutrophil mediated immunity	2.44E−21	3.68E−18
Hypo-methylated	TSS1500	GO:0042119	Neutrophil activation	4.16E−21	4.70E−18
Hypo-methylated	TSS1500	GO:0036230	Granulocyte activation	7.50E−21	6.78E−18
Hypo-methylated	TSS200	GO:0002446	Neutrophil mediated immunity	5.50E−25	1.89E−21
Hypo-methylated	TSS200	GO:0036230	Granulocyte activation	2.75E−24	4.72E−21
Hypo-methylated	TSS200	GO:0002283	Neutrophil activation involved in immune response	6.79E−24	5.84E−21
Hypo-methylated	5′UTR	GO:0002446	Neutrophil mediated immunity	4.53E−19	1.10E−15
Hypo-methylated	5′UTR	GO:0002283	Neutrophil activation involved in immune response	8.81E−19	1.10E−15
Hypo-methylated	5′UTR	GO:0043312	Neutrophil degranulation	8.81E−19	1.10E−15
Hypo-methylated	5′UTR	GO:0042119	Neutrophil activation	1.33E−18	1.25E−15
Hypo-methylated	5′UTR	GO:0036230	Granulocyte activation	2.01E−18	1.51E−15
Hypo-methylated	1stExon	GO:0002446	Neutrophil mediated immunity	1.28E−20	3.48E−17
Hypo-methylated	1stExon	GO:0002283	Neutrophil activation involved in immune response	3.38E−20	3.48E−17
Hypo-methylated	1stExon	GO:0043312	Neutrophil degranulation	3.38E−20	3.48E−17
Hypo-methylated	1stExon	GO:0042119	Neutrophil activation	4.97E−20	3.84E−17
Hypo-methylated	1stExon	GO:0036230	Granulocyte activation	7.27E−20	4.50E−17
Hypo-methylated	Body	GO:0042119	Neutrophil activation	7.07E−14	1.49E−10
Hypo-methylated	Body	GO:0036230	Granulocyte activation	1.09E−13	1.49E−10
Hypo-methylated	Body	GO:0002446	Neutrophil mediated immunity	1.41E−13	1.49E−10

*TSS* transcription start site, *TSS1500* within 1500 bps of a TSS, *TSS200* within 200 bps of a TSS, *UTR* untranslated region, *1stExon* the first exon of a gene

Although the DMGs based on different gene region CpGs are quite different, there are some overlap genes between each region DMGs and DEGs as shown in Fig. 3c, revealing that different DEGs have different methylation-altered regions. In order to explore the relationship between methylation level and expression level of the overlap genes in different regions, we have classified the overlap gene to four classes: hyper-methylated and downregulated group (hyper-down), hypo-methylated and upregulated group (hypo-up), hyper-methylated and upregulated group (hyper-up), and hypo-methylated and downregulated group (hypo-down). As Fig. 3d shows, the number of hypo-up group genes is significantly bigger than other group in each gene region, indicating hypo-methylation might be a key altered epigenetic modification in PD patients. The hypo-up genes for each region are in supplementary materials (Additional file 1: Table S7).

### Identification of blood-based DMGs in PD based on all region CpGs

**Fig. 4:**
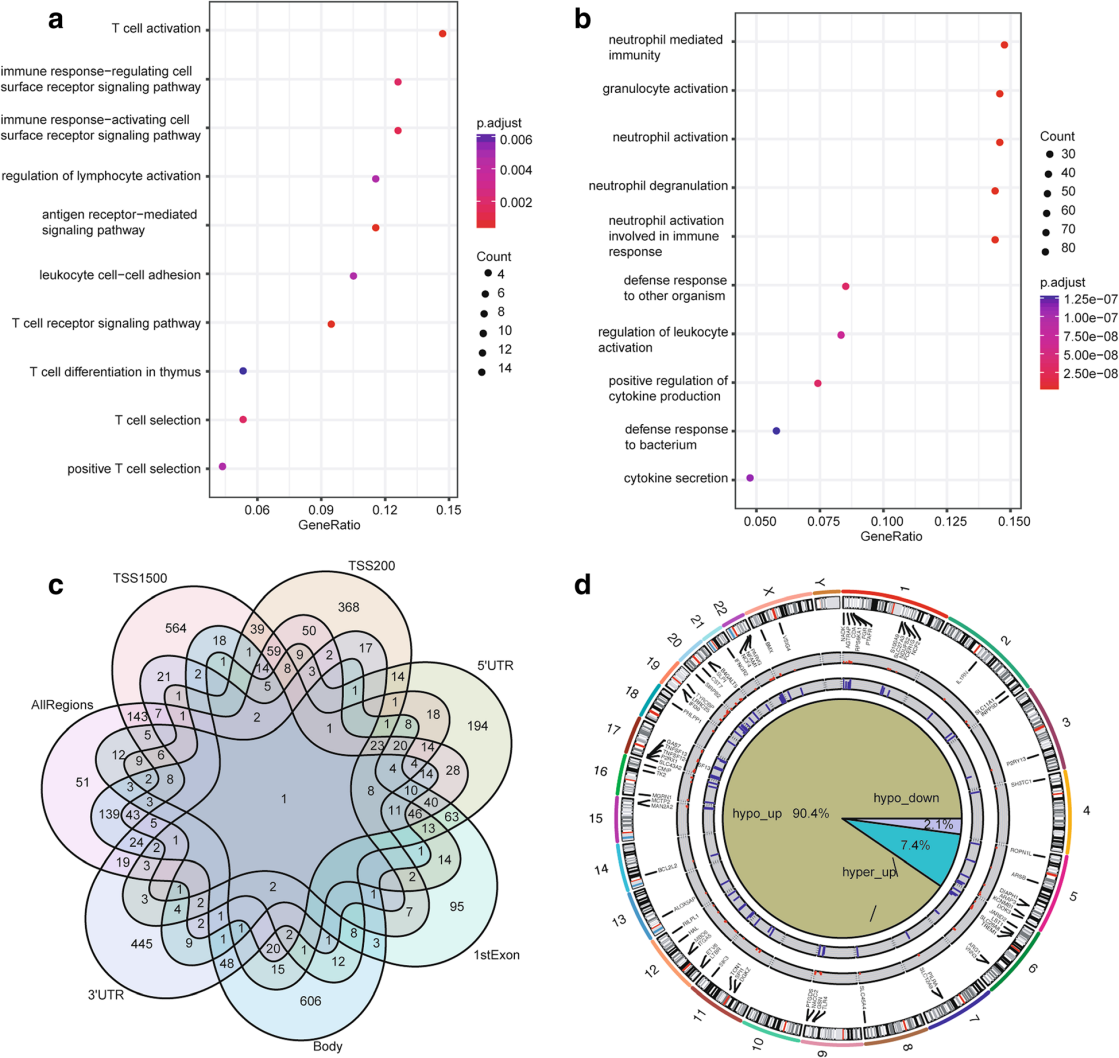
Enrichment analysis results and characteristics of DMGs based on all region CpGs. **a** Hyper-methylated genes enrichment analysis dotplot. The *x*-axis is the gene ratio. The *y*-axis is the enriched term list. The dot size represents the number of genes associated with a specific term. The dot color represents the adjusted *p* value of GSEA. **b** Hypo-methylated genes enrichment analysis dotplot. **c** Venn plot for different region DMGs and all region DMGs. The numbers on the diagram represent the DMG numbers in a specific region or multiple regions. Each region name is labeled beside the region circle. **d** Hypo-up genes genome position. The inner track is a pie chart for different overlap groups. Hypo-up represents hypo-methylated and upregulated genes. Hyper-up represents hyper-methylated and upregulated genes. Hypo-down represents hypo-methylated and downregulated genes. The second track is the barplot for the delta of beta value of hypo-up genes. The third track is the barplot for log2FC of hypo-up genes. The fourth track is parts of hypo-up gene names. The fifth track is the link from hypo-up gene name to chromosome position. The outer track is each chromosome

In order to discover DMGs regardless of the methylation region, we used all gene region-associated CpGs to measure gene methylation level and we identified 891 significantly differentially methylated genes, which contain 125 hyper-methylated genes and 766 hypo-methylated genes (Additional file 1: Table S8). Hyper-methylated genes are associated with following BP terms: T cell activation, immune response-regulating cell surface receptor signaling pathway, and regulation of lymphocyte activation; KEGG pathways: natural killer cell mediated cytotoxicity, Th17 cell differentiation, T cell receptor signaling pathway, etc. Hypo-methylated genes are associated with following BP terms: neutrophil mediated immunity, granulocyte activation, neutrophil activation, etc.; CC terms: vesicle lumen, cytoplasmic vesicle lumen, secretory granule membrane, etc.; MF terms: carbohydrate binding, cytokine receptor binding, growth factor binding, etc. (Fig. 4a–b and Additional file 1: Table S9). The Venn diagram for DMGs from all regions and specific region is shown in Fig. 4c. Notably, there are only 51 DMGs without intersection with DMGs based on specific region CpGs. There are 139 unique overlap DMGs between DMGs based on all region CpGs and DMGs based on body region CpGs. For these overlap DMGs, their dominant methylation-altered region is the gene body region. There are 143 unique overlap DMGs between DMGs based on all region CpGs and DMGs based on TSS1500 region CpGs. As for these overlap DMGs, their dominant methylation-altered CpGs are at TSS1500 region. Notably, the unique overlap numbers between DMGs based on all region CpGs and DMGs based on the other four gene region CpGs (TSS200, 5′UTR, 1stExon, 3′UTR) are less than 20, which indicates that genes with the dominant methylation-altered region at TSS200, 5′UTR, 1stExon and 3′UTR are relatively less than that at TSS1500 and gene body region. We also checked the relationship between DMGs based on all region CpGs and DEGs. As Fig. 4d shows, 90.4% overlap genes are hypo-methylated and upregulated genes. These upregulated genes may play important roles in initiation or progression of PD. In order to explore the relationship between these hypo-up genes, we examined their genome location relationship. As Fig. 4d shows, chromosome 1, chromosome 11, and chromosome 17 are enriched with these hypo-methylated and upregulated genes. In addition, we also identified these hypo-up gene-associated CpGs that are significantly differentially methylated between PD patients and healthy controls (Additional file 1: Table S10), in which cg13060970, cg02861056, cg21495704, cg16643542, cg19081101, etc., have been demonstrated to be associated with PD [19].

**Fig. 5:**
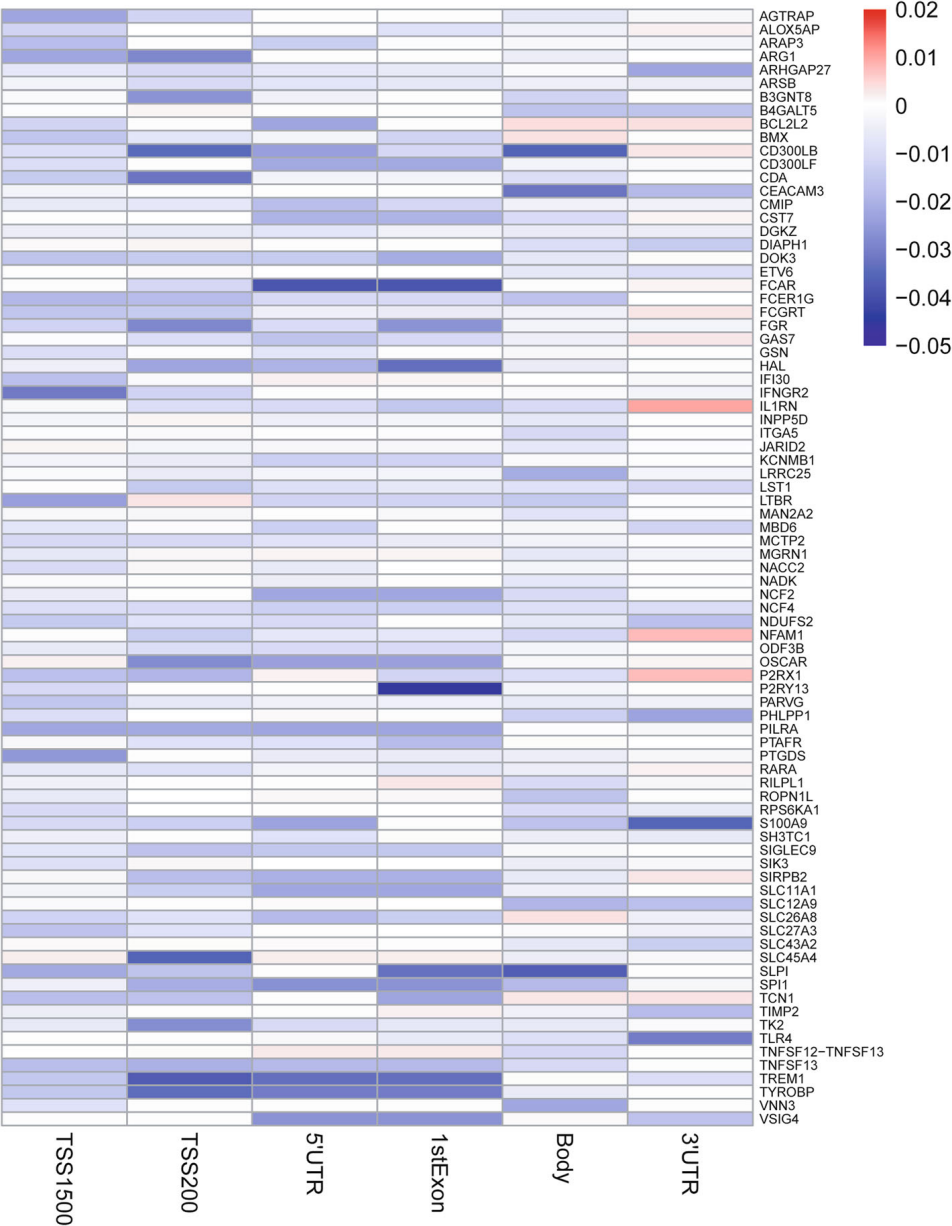
Hyper-up gene delta of beta value for each region. The columns are each region and the rows represent each hypo-up gene. The values are delta of beta value between PD patients and healthy controls at specific region for specific gene

In order to make it clear which dominant hypo-methylated region leads to the upregulation of these 85 hypo-up genes, we used the delta of beta value of each region between PD and control to determine the dominant hypo-methylated regions (Additional file 1: Table S11). Moreover, we randomly took 200 PD patients and 200 healthy controls from both gene expression data and gene methylation data. The Pearson correlation test was used to check the correlation between gene expression and gene methylation level of different regions. Interestingly, we found that 74% of the correlation coefficient is negative and 52 genes with significant correlation between gene expression and methylation level of some regions. The detailed information of correlation coefficient, *p* value and significantly correlated regions is shown in Table S12. In addition, we made a heat map (Fig. 5) of which the rows are hypo-up genes, the columns are different gene regions and the values are delta of beta values. As the heat map shows, different hypo-up genes have different dominant hypo-methylated regions. For example, the dominant hypo-methylated region for P2RY13 is 1stExon. And some genes have multiple dominant hypo-methylation regions, such as FCAR with two dominant hypo-methylated regions 5′UTR and 1stExon. In addition, the heat map also shows that each gene region can be the dominant hypo-methylated region of specific gene. Therefore, we can use region specific, all regions, or dominant methylation-altered region to measure gene methylation according to our research goals.

### Classifier construction

**Fig. 6:**
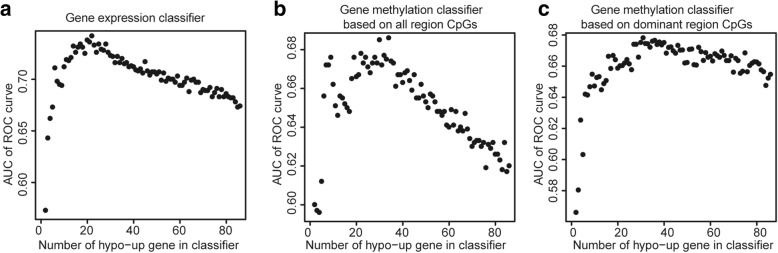
Scatter plots illustrating the relationship between prediction power and number of hypo-up genes in classifier. **a** Gene expression classifier. *x*-axis represents the number of hypo-up genes in the classifier and *y*-axis represents the AUC value of the ROC curve for the classifier. AUC stands for area under the curve. ROC stands for receiver operating characteristic. **b** Gene methylation classifier based on all region CpGs. **c** Gene methylation classifier based on dominant methylation-altered regions

**Fig. 7:**
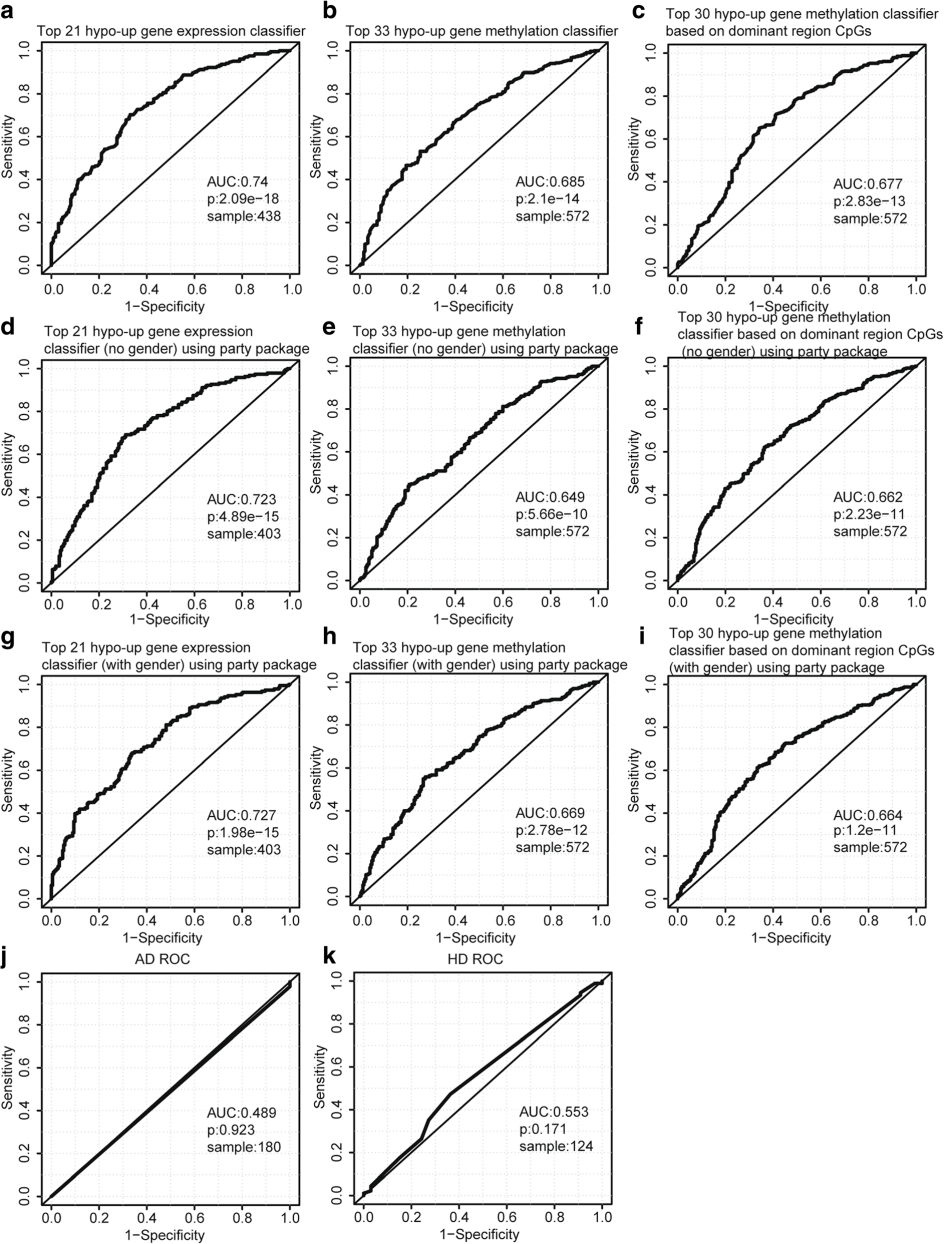
ROC curves for hypo-up gene classifiers. **a** Top 21 hypo-up gene expression classifier. AUC stands for area under the curve. ROC stands for receiver operating characteristic. The *p* value is calculated using the “wilcox.test” function. **b** Top 33 hypo-up gene methylation classifier based on all gene region CpGs. **c** Top 30 hypo-up gene methylation classifier based on dominant methylation-altered region CpGs. **d–i** These random forest classifiers composed of conditional inference trees are implemented by “party” package. **d** Top 21 hypo-up gene expression classifier for 403 samples with gender information but does not consider gender information as an input feature. **e** Top 33 hypo-up gene methylation classifier based on all gene region CpGs without “gender” as a feature. **f** Top 30 hypo-up gene methylation classifier based on dominant methylation-altered region CpGs without “gender” as a feature. **g** Top 21 hypo-up gene expression classifier with “gender” as a feature. **h** Top 33 hypo-up gene methylation classifier based on all gene region CpGs with “gender” as a feature. **i** Top 30 hypo-up gene methylation classifier based on dominant methylation-altered region CpGs with “gender” as a feature. **j** The ROC curve of top 21 gene expression classifier for AD samples. **k** The ROC curve of top 21 gene expression classifier for HD samples

Random forest algorithm and leave-one-out cross validation method were used to construct three classifiers to distinguish PD patients from healthy controls based on the blood gene expression data and methylation data of the 85 hypo-up genes. Then, the average importance of each hypo-up gene for each classifier was calculated using the “importance” function of “RandomForest” package, and the hypo-up genes were ranked in descending order based on their importance (Additional file 1: Table S13). Subsequently, in order to identify the best predictors of each classifier, we added these hypo-up genes to each classifier one by one in order of the importance rank. The prediction power of these top genes is shown as Fig. 6. We found that top 21, top 33, and top 30 are the best predictors of the three classifiers, respectively, which contain in total 53 hypo-up genes (Additional file 1: Table S14). The ROC curves of the three classifiers with best predictors are shown in Fig. 7a–c. As Fig. 7a–c shows, the classifier based on gene expression data has a good prediction power (AUC: 0.74, *p* value: 2.09e−18). Two classifiers are based on gene methylation data, one based on all gene region CpGs, and one based on the dominant methylation-altered regions. The prediction powers of the two methylation classifiers are similar (AUC, 0.685 and 0.677; *p* value, 2.1e−14 and 2.83e−13). Using dominant methylation-altered region to measure gene methylation level, we can use relatively less genes to obtain similar prediction power. Additionally, we have constructed another three random forest classifiers with these best predictors which are composed of conditional inference trees [46]. The ROC curves of these three classifiers are shown in Fig. 7d–f. The gene expression classifier still has the best prediction power (AUC, 0.723; *p* value, 4.89e−15). The methylation classifier based on dominant methylation-altered region CpGs has a relatively higher prediction power (AUC, 0.662; *p* value, 2.23e−11) than that based on all region CpGs (AUC, 0.649; *p* value, 5.66e−10). Then, we checked whether adding gender information to the classifiers will improve their prediction power. As Fig. 7g–i shows, each classifier has a relatively higher prediction power (AUC, 0.727, 0.669 and 0.664; *p* value, 1.98e−15, 2.78e−12, and 1.2e−11) than the corresponding classifier without gender information (Fig. 7d–f). In addition, we have validated the PD specificity of our gene expression classifier by using two different protein aggregation disease datasets: one Alzheimer’s disease (GSE85426) and one Huntington’s disease expression data set (GSE51799). The AUCs of ROC of the two test datasets are shown in Fig. 7j, k (AUC, 0.489 and 0.553; *p* value, 0.923 and 0.171), which indicates that our expression classifier has no prediction power for Alzheimer’s disease and Huntington’s disease, but is efficient and specific for Parkinson’s disease. The quality of the classifiers, to some extent, reveals that the best predictors from these hypo-up genes could be potential blood biomarkers for PD.

## Discussion

In our study, we identified blood-based signatures in PD, and we found that dysregulated genes are mainly associated with the structural constituent of cytoskeleton, immune system, phagosome, and lysosome pathways. The structural constituent of cytoskeleton was proven to be associated with dopaminergic neurotransmission [38], and activation of the innate immune system has been verified in association with or in response to Lewy body formation [41, 42], suggesting that signature genes can participate in PD pathology. In addition, phagosome and lysosome pathway play important roles in mis-folded protein degradation [39]. Decrease of dopaminergic neurotransmission, mis-folded protein aggregation, and Lewy body formation are the well-known pathological hallmarks of PD [40, 47]. The blood signatures identified by our methods are associated with well-known PD hallmarks, indicating that blood biomarkers for PD is feasible.

As previous studies mainly focused on promoter region methylation [16–18] and some PD-associated CpGs do exist at other gene regions [19, 20], we measured gene methylation level based on different gene region CpGs and all gene region CpGs. We used both *M* value and beta value to perform the differential analysis, as the former has better statistical power and the latter has better biological interpretation. We found that 5′UTR and 1stExon region share many DMGs, while the other four regions share few DMGs with other regions, revealing that the methylation alteration between PD and control at 5′UTR and 1stExon is much more similar than other regions. Although most DMGs based on all region CpGs can be identified by the specific region analysis, there are some unique DMGs identified based on all region CpGs. Therefore, by integration of region-specific analysis and all region analysis, we can identify relatively comprehensive DMGs and we can find some region-specific DMGs. Moreover, the enrichment analysis for DMGs based on different region CpGs or all region CpGs revealed that these DMGs are also associated with some PD hallmarks, such as innate immunity-associated GO terms, which are associated with the formation of Lewy body [41, 42] and T cell activation, which suggests an inflammatory response [45]. These results were revealed using different or all regions CpGs to measure gene methylation levels that could find some PD-associated molecules.

We observed some overlap genes between DEGs and DMGs, although the data are from different samples and obtained with different analysis methods. Interestingly, the majority of overlap genes between DMGs and DEGs are hypo-methylated and upregulated genes (hypo-up), which indicates that hypo-methylation might be a key PD-associated epigenetic modification and hypo-methylation of some PD-associated genes will lead to upregulation of these genes. For example, the hypo-methylation of SNCA promoter region, will lead to the upregulation of SNCA [14], and then lead to the aggregation of α-synuclein and the formation of Lewy bodies [48]. There are some verified PD-associated genes in the hypo-up gene list, such as ARG1, of which the upregulation is one phenomenon of the alterative activation states of microglia, and microglia-mediated neuroinflammation is a hallmark of PD [49]. ARHGAP27 is another already established PD gene [50]. ARHGAP27 encoded protein plays a role in clathrin-mediated endocytosis. In addition, FCER1G, another hypo-up gene, is upregulated in microglia in PD patients [51]. Other work previously reported that GPR97 is upregulated in the blood of PD patients and regarded GPR97 as a blood signature of PD [52]. IL1RN and MCTP2 are proved to be associated with PD by meta-analysis [53, 54]. These known PD signatures, to some extent, reveal the feasibility of our analysis. Therefore, PD samples with both gene expression data and gene methylation data from the same individual person are in need. This will facilitate the process of integration analysis and the study of the relationship between the dysregulated genes and their associated methylation alteration, and it will be beneficial to study the pathology of PD. In addition, the collection of information concerning other risk factors (age, gender, ethnicity, family history, etc.) for PD is also very important, which will provide important features for the classifier and might improve the prediction power. Moreover, in future studies, we will add PD brain samples to our analysis and attempt to identify PD-associated molecules either in brain tissues or in blood.

We also wanted to determine which dominant methylation-altered region will lead to hypo-methylation and upregulation of these hypo-up genes. We found that different genes have different dominant hypo-methylated regions. So, only using one specific region CpGs to measure the gene level might miss some PD-associated DMGs. Therefore, we believe our analysis can identify more PD-associated DMGs. As gene expression data and methylation data are from different samples, the findings from different datasets suggest a type of independent validation.

Finally, we used the hypo-up gene as features to construct three classifiers—one based on gene expression data, and two based on gene methylation data (all region CpGs and dominant methylation-altered region CpGs). The three classifiers all have good prediction power. Using our classifier to diagnose PD would only require blood samples from patients and quantification of the gene expression or methylation level of these best predictors, and then our classifier will give a prediction about the probability of the person suffering from PD. If gene expression data and DNA methylation data are from the same samples, we could integrate the gene expression classifier and DNA methylation classifier to improve the prediction power of our classifiers. Eventually, we believe that the best predictors from these hypo-up genes could be the potential blood biomarkers for PD, which might benefit the early-stage diagnosis or the future prevention or treatment of PD.

## Conclusions

Our study, for the first time, integrated gene expression and DNA methylation data based on different gene region CpGs, all gene region CpGs, and dominant methylation-altered region CpGs. We found hypo-methylation as a key epigenetic modification for PD in blood samples from patients. Furthermore, we identified a blood signature for PD composed of 53 hypo-up genes.

## Additional file

Additional file 1: **Table S1.** Differentially Expressed Gene List. **Table S2.** Previous differential probes and our analysis results. **Table S3.** Differentially expressed gene functional enrichment results. **Table S4.** Differentially methylated intergenic CpG sites. **Table S5.** Differentially methylated gene list. **Table S6.** Gene set enrichment analysis results (GO-BP). **Table S7.** Hypo-up genes in different regions. **Table S8.** Differentially methylated gene list based on all region CpGs. **Table S9.** Gene enrichment analysis results. **Table S10.** Hypo-up gene-associated differentially methylated CpGs. **Table S11.** Dominant methylation-altered regions for hypo-up genes. **Table S12.** Correlation Coefficient between gene expression and region-specific methylation level. **Table S13.** The importance of each hypo-up gene in gene expression classifier. **Table S14.** Union of best predictors of the three classifiers. (ZIP 1455 kb)

## Abbreviations

AD: Alzheimer’s disease
AUC: Area under the curve
BH: Benjamini and Hochberg’s method
BP: Biological process
CC: Cellular component
CpG: 5′—C—phosphate—G—3′
DEG: Differentially expressed gene
DMG: Differentially methylated gene
GEO: Gene expression omnibus
GSEA: Gene set enrichment analysis
HD: Huntington’s disease
Hyper-down: hyper-methylated and downregulated
Hyper-up: hyper-methylated and upregulated
Hypo-down: hypo-methylated and downregulated
Hypo-up: hypo-methylated and upregulated
KEGG: Kyoto Encyclopedia of Genes and Genomes
MF: Molecular function
PD: Parkinson’s disease
ROC: Receiver operating characteristic curve
